# The Contribution of Race to Breast Tumor Microenvironment Composition and Disease Progression

**DOI:** 10.3389/fonc.2020.01022

**Published:** 2020-06-30

**Authors:** Gina Kim, Jessica M. Pastoriza, John S. Condeelis, Joseph A. Sparano, Panagiota S. Filippou, George S. Karagiannis, Maja H. Oktay

**Affiliations:** ^1^Department of Anatomy and Structural Biology, Montefiore Medical Center, Einstein College of Medicine, Bronx, NY, United States; ^2^Integrated Imaging Program, Montefiore Medical Center, Einstein College of Medicine, Bronx, NY, United States; ^3^Department of Surgery, Montefiore Medical Center, Einstein College of Medicine, Bronx, NY, United States; ^4^Gruss-Lipper Biophotonics Center, Montefiore Medical Center, Einstein College of Medicine, Bronx, NY, United States; ^5^Department of Medicine (Oncology), Montefiore Medical Center, Einstein College of Medicine, Bronx, NY, United States; ^6^School of Health & Life Sciences, Teesside University, Middlesbrough, United Kingdom; ^7^National Horizons Centre, Teesside University, Darlington, United Kingdom; ^8^Department of Pathology, Montefiore Medical Center, Einstein College of Medicine, Bronx, NY, United States

**Keywords:** breast cancer, tumor microenvironment, breast cancer racial disparity, breast cancer outcome, tumor microenvironment of metastasis (TMEM)

## Abstract

Breast cancer is the second most commonly diagnosed cancer in American women following skin cancer. Despite overall decrease in breast cancer mortality due to advances in treatment and earlier screening, black patients continue to have 40% higher risk of breast cancer related death compared to white patients. This disparity in outcome persists even when controlled for access to care and stage at presentation and has been attributed to differences in tumor subtypes or gene expression profiles. There is emerging evidence that the tumor microenvironment (TME) may contribute to the racial disparities in outcome as well. Here, we provide a comprehensive review of current literature available regarding race-dependent differences in the TME. Notably, black patients tend to have a higher density of pro-tumorigenic immune cells (e.g., M2 macrophages, regulatory T cells) and microvasculature. Although immune cells are classically thought to be anti-tumorigenic, increase in M2 macrophages and angiogenesis may lead to a paradoxical increase in metastasis by forming doorways of tumor cell intravasation called tumor microenvironment of metastasis (TMEM). Furthermore, black patients also have higher serum levels of inflammatory cytokines, which provide a positive feedback loop in creating a pro-metastatic TME. Lastly, we propose that the higher density of immune cells and angiogenesis observed in the TME of black patients may be a result of evolutionary selection for a more robust immune response in patients of African geographic ancestry. Better understanding of race-dependent differences in the TME will aid in overcoming the racial disparity in breast cancer mortality.

## Introduction

Breast cancer is the second most common cancer in women in the U.S. following skin cancer, and is the second leading cause of cancer death (1, 2). In both female and male breast cancer, black race or African American (AA) ethnicity is associated with a worse prognosis compared to white race or European American (EA) ethnicity (3–6). Clinical and treatment factors associated with worse outcomes for black race in breast cancer are well-described (Table 1). Although breast cancer incidence and mortality have declined by ~40% in the U.S. between 1989 and 2017 (2), mortality rates have declined less in black women, which has contributed to persistently higher breast cancer mortality rate for black women (17). Furthermore, despite the lower incidence rate, the death rate for black women with breast cancer is now 40% higher than for white women (1, 2). For black women younger than 50 years of age, the death rate is double than that of white women of the same age group (2).

**Table 1:** Factors contributing to worse clinical outcomes for black race in breast cancer.

**Clinical presentation**
More advanced stage disease (7) Higher rates of triple-negative disease (8) Higher rates of obesity (9)
**Treatment**
Poorer adherence to chemotherapy (10) and endocrine therapy (11) Higher rates of taxane neuropathy (12)
**Other factors**
Worse outcomes in ER-positive breast cancer despite comparable therapy (9, 13–15) More comorbidities and disparities in access to care (16)

A widening racial gap in survival has also been observed for women in the US Department of Defense healthcare system (18), as well as for women undergoing NCI-sponsored clinical trials receiving contemporary therapy (Table 2), suggesting that factors other than disparities in care may be playing a role in contributing to inferior outcomes (20). A similar disparity in survival was also observed in patients with ER+/HER2- disease treated at Montefiore Medical Center, which serves a large African American population (13).

**Table 2:** Adjuvant Breast Cancer Trials.

**Study/Cohort**	**No**.	**Black**	**Stage**	**Black race and risk of recurrence**
E1199 (NCT00004125) (9)	4,819	405 (8.4%)	II–III	↑ 1.58-fold (*p* = 0.002) in ER+/HER2- disease (self-identified race)
E5103 (NCT00433511) (14, 15)	4,994	568 (11.4%)	II–III	↑ 1.5-fold (*p* = 0.027) in ER+/HER2- disease (in subset with genetic African-American [*n* = 386] or European-American [*n* =2,473] by ancestry informative markers)
TAILORx (NCT00310180) (19)	9,223	722 (7.8%)	I–II	↑ 1.29-fold (*p* = 0.02) in entire population, and 1.8-fold (*p* <0.001) for 21 gene RS −11 to 25
Montefiore-Einstein cohort (13)	3,890	1,393 (35.4%)	I–III	↑ 1.84-fold (*p* < 0.05) in ER+/HER2- disease (self-identified race)

*↑, Increased*.

Indeed, several studies have indicated that racial disparity in breast cancer outcome between patients of African compared to those of Caucasian ancestry are due to biological factors including differences in gene expression patterns of tumor cells as well as differences in the local milieu (or context) in which cancer cells reside, typically referred to as the tumor microenvironment (TME) (21). TME encompasses a variety of cells including fibroblasts, adipocytes, immune cells, endothelial cells, as well as a plethora of signaling molecules and extracellular matrix (ECM) components. The non-cancerous stromal cells influence the behavior of cancer cells by direct contact, as well as by secreting ECM proteins, chemokines, cytokines and growth factors. Thus, it is the dynamic interplay between cancer cells, non-cancerous cells and other components of TME that dictates the growth and invasiveness of tumors and may contribute to racial disparity in breast cancer outcome. This review will focus on the racial disparities in TME as potential modulators of cancer progression, metastasis and response to therapy.

## Racial/Ethnic Disparities in the Breast Cancer Microenvironment

Breast cancer is an extremely heterogeneous disease at multiple levels, including histologic subtype, grade, hormone and growth factor receptor status, as well as gene expression pattern (22). Molecular profiling based on the analysis of gene copy number, mRNA, microRNA and protein expression supports at least four (23), and up to ten intrinsic subtypes (24). Although an association of these intrinsic subtypes with disease outcome has been clearly demonstrated (22), it has been increasingly appreciated that the tumor microenvironment (TME) also plays an important role in regulating breast cancer biology at all stages of progression and ultimately influences disease outcome (25). Moreover, multiple lines of evidence indicate that black patients exhibit a TME with more pronounced pro-tumorigenic properties, which may be responsible for, and contribute to the disparity in breast cancer survival.

### Disparity in Breast Cancer Immune Landscape

A number of immune cells reside within the TME and contribute to cancer progression. Among the well-studied ones are tumor-associated lymphocytes (TILs), regulatory T cells (T-regs), neutrophils, tumor-associated macrophages (TAMs), and myeloid-deprived suppressor cells (MDSCs).

#### Lymphocytes

TILs, the most abundant immune cells within breast TME, convey a good prognosis especially in patients with triple negative (TN) disease (26–28). In particular, high TIL count in TN disease has been associated with better survival, as well as better response to treatment (29, 30). Although the analysis of gene expression variants have shown higher expression of genes associated with immune response in tumors from African American (AA) compared to European American (EA) patients (31), the comparison of TIL counts, either as percent-area of stroma, or as percent-area of the whole section did not show any differences between these two racial groups (32). Likewise, the distribution of tumors that were lymphocyte-predominant (>50% TIL), lymphocyte-moderate (10–50% TIL) and lymphocyte-poor (<10% TILs) was not significantly different (32). The immunomodulatory score (33), which helps predict response to neoadjuvant chemotherapy, was also not different between AA and EA patients (32).

Unlike TILs, increased number of T-regs in breast TME has been associated with decreased relapse-free and overall survival (34, 35). This is not surprising as T-regs are suppressors of T cell responses and mediators of immune tolerance, and as such, T-regs may contribute to immune evasion of cancer cells. Indeed, ablation of T-regs leads to CD4 T-cell- and interferon-γ (INF-γ)-dependent reduction of primary and metastatic tumor growth in a transgenic mouse model of breast cancer (36). When analyzed as relative proportion among 9 immune cell populations (B-cells, dendritic cells, eosinophils, macrophages, mast cells, neutrophils, NK cells, CD4 and CD8 T-cells), T-regs were present in significantly higher proportion in TME of AA than of EA patients (32). It is therefore possible that more aggressive disease in AA compared to EA may be due to more pronounced immunosuppressive TME in breast cancers of AA patients. Since the recruitment of T-regs into the TME occurs partly via the C-X-C motif chemokine-12 (CXCL12) signaling factor, it would be interesting to see if TME in breast cancers from AA compared to EA patients produces more CXCL12.

#### Myeloid Cells

Neutrophils have been typically involved in the pathophysiology of acute infection and elimination of bacteria. However, about a decade ago, studies in pre-clinical models of cancer demonstrated that depending on the levels of chemokines in TME, tumor-associated neutrophils (TANs) may develop an either pro- or anti-tumor phenotype (37). More recent meta-analyses showed that a high neutrophils-to-lymphocytes ratio is associated with worse outcome (38–40). Neutrophils are also found to be potent suppressors of T-cell mediated immunity (41). Moreover, neutrophils can expulse their DNA to create so-called neutrophil extracellular traps (NETs), which can promote metastasis (42). Relative to other immune cell populations, the mean proportion of neutrophils was not found to be different between AA and EA (32). Quite interestingly, up to 12.5% of healthy women of AA descent were found to have neutropenia (43). However, it is not yet clear how that may affect breast cancer incidence and progression.

The role of TAMs in the progression of breast cancer has been extensively studied (44–47). This is not surprising given that macrophages are the most abundant leukocytes in breast TME in both AA and EA patients (32). Macrophages are extremely plastic and under constant influence of TME, which can modify them to function as either tumor inhibitory (M1) or tumor promoting (M2) agents (48, 49). M1 macrophages, also called classically-activated macrophages, secrete pro-inflammatory cytokines such as INF-γ, TNF-α, IL-1, IL6, and IL-12, while M2 macrophages secrete anti-inflammatory cytokines, such as IL-10 and TGF-β. It is important to mention that both within and between the M1 and M2 polarization states, there exist several subcategories of macrophage phenotypes. Although most studies associate high macrophage density with poor outcome (50–52), macrophage density does not seem to play a role in the outcome of patients who have ER+ tumors smaller than 1 cm (53). However, TAMs seem to differ in TME of AA and EA patients not only in their density, but also in their composition. For instance, AA patients compared to EA and non-black Hispanic patients tend to have not only higher macrophage density (54), but also higher density of pro-tumorigenic M2, CD206-expressing macrophages in the TME (55). Consistent with the well-established role of M2 macrophages in promoting tumor invasion, angiogenesis, metastasis and immunosuppression (56–58), the density of CD206 M2 macrophages was found to be a significant predictor of progression-free survival independently of race (54). This even held true after adjusting for race and HER2 expression. Interestingly, if evaluated as a mean proportion of the leukocyte compartment within TME, tumors from AA compared to EA patients have a higher overall macrophage score, but tumors from EA patients score higher for M2 macrophages (32). One of the pro-tumorigenic properties of M2 macrophages is their ability to promote angiogenesis (58, 59). Indeed, M2 TAMs secrete various cytokines as well as matrix degrading enzymes that orchestrate not only cancer cell invasion, but also angiogenesis. In particular, a subset of M2 macrophages that expresses the tyrosine kinase receptor Tie2 produces large amounts of vascular endothelial growth factor (VEGF), which, in turn, regulates cancer cell dissemination (60, 61). Thus, macrophages serve as principal modifiers and regulators of blood vessel development and structure in the tumor microenvironment, suggesting that racial disparities in macrophage populations may indirectly shape the angiogenic milieu in different ethnic groups.

Myeloid-derived suppressor cells (MDSCs) represent well-established mediators of the immunosuppressive tumor microenvironment, and also serve as critical regulators of angiogenesis, cancer cell invasion and migration, as well as pre-metastatic niche formation (62–64). MDSCs are currently categorized into two distinct subtypes with clearly defined surface phenotype and functions, the granulocytic (G-MDSC) and the monocytic (Mo-MDSC) types (65).The levels of G-MDSCs in the peripheral blood of breast cancer patients receiving neo-adjuvant chemotherapy (doxorubicin and cyclophosphamide) are significantly elevated, especially for those who do not present with pathologic complete response (pCR) (66). Interestingly, this study additionally demonstrated that AA patients present with a comparably lower increase in G-MDSC levels following chemotherapy, compared to Caucasians (66).These observations suggest that racial disparities in MDSC responses and functions in the breast TME, especially in the context of chemotherapy treatment, may account for significant differences in tumor progression and even therapeutic outcome among different ethnic backgrounds.

### Disparity in Breast Cancer Vascular Compartment

Microvascular density has been consistently associated with tumor progression and outcome in breast cancer (67), because blood vessels are critical for the development and progression of the primary breast cancer, cancer cell dissemination to distant sites (60, 68, 69), metastatic seeding, as well as outgrowth of metastatic nodules. Angiogenesis is a complex process regulated by a plethora of cytokines produced in response to hypoxia-induced activation of HIF-1 transcription factors (70). Given such importance of vasculature in tumor progression, it is not surprising given the disparity in outcome described above that one of the most striking differences in TME between patients of African and European ancestry is in the biology of angiogenesis (55). A comprehensive study by Martin et al. (55) looked at differences in gene enrichment in specific biological processes in tumor stroma and tumor epithelium, separated by laser capture micro-dissection, between black and white patients. The study found that patients of African ancestry had significantly higher expression of genes involved in cell cycle control and chemotaxis in tumor epithelium, while tumor stroma was enriched for genes involved in neovascularization. This study also found increased microvascular density in TME from AA patients. Interestingly however, an analysis of the National Cancer Database (NCDB) found that black race was not associated with higher risk of lymphovascular invasion in patients with early ER+/HER2- breast cancer (71). Given that a subset of TAMs stimulates angiogenesis, it is plausible that breast cancers in black patients release more macrophage chemotactic signals such as CSF-1, which could result in macrophage recruitment, increased density of proangiogenic TAMs and subsequent increase in microvascular density. Indeed, plasma levels of granulocyte colony-stimulating factor (G-CSF) was found to be elevated in African-American compared with Caucasian patients (72). Increased microvascular density along with increased density of proangiogenic CD206 expressing macrophages within TME likely contribute to an enhanced assembly of specialized doorways for cancer cell dissemination to distant sites called tumor microenvironment of metastasis (TMEM) doorways (60, 61). TMEM doorways are sites of localized transient vascular permeability. Each TMEM doorway is composed of one proangiogenic CD206 macrophage expressing high levels of Tie2 receptor, one tumor cell expressing high levels of actin regulatory protein Mena, and one endothelial cell expressing angiopoietin-2 (Ang2), all in direct physical contact (60). The TMEM doorway is a clinically validated prognostic biomarker for breast cancer metastasis to distant sites such as lung, bone or brain (73–75). It would be interesting to investigate if the density of TMEM doorways differs in breast TME of patients from different racial ancestry and if the difference in TMEM density contributes to the disparity in breast cancer outcome.

### Cancer-Associated Fibroblasts (CAFs), Extracellular Matrix (ECM), and Breast Density in Patients of Different Racial/Ethnic Backgrounds

Cancer-associated fibroblasts (CAFs) are the most common stromal cell type of mesenchymal origin in the tumor microenvironment (76). However, due to the lack of molecular markers specific for CAFs, it is challenging to identify and study them (77). Nevertheless, it has been observed that breast CAFs secrete a large number of growth factors such as fibroblast growth factor (FGF), transforming growth factor beta (TGF-β), CXCL12, and hepatocyte growth factor (HGF), as well as various cytokines that contribute to cancer cell proliferation, invasiveness and angiogenesis (76). While, to the best of our knowledge, the effect of CAFs on cancer progression from patients of different racial backgrounds has not been investigated, one study describes the effect of ECM and fibroblasts isolated from healthy pre-menopausal women of various racial backgrounds on breast cancer cell growth and invasion both *in vivo* and *in vitro* (78). This study reports that fibroblasts from both AA and EA women enhanced cancer progression albeit in slightly different ways. *In vitro*, ECM from AA women induced invasiveness of TN cancer cells, while fibroblasts from EA women induced invasiveness of ER+/PR+ cancer cells. In xenograft models, ECM from EA women increased tumorigenesis of ER+/PR+ cells and enhanced metastasis. However, *in vitro* studies must be viewed with caution since *in vitro* assays suffer from uncertainty regarding the lack of TME associated factors which can lead to the observation of cell phenotypes that are unrelated to cell behavior *in vivo*.

According to several studies it seems that single nucleotide polymorphisms (SNPs) in the FGF family of genes may influence the risk for breast cancer in patients of various racial backgrounds. In particular, SNP variants in the fibroblast growth factor receptor-2 (FGFR-2) gene and/ or the FGFR-2 promoter are associated with an increased risk of breast cancer in Chinese women (79, 80), Northern Indian (81), Caucasian (82), and AA women (83). Thus, it is plausible that there are racial differences in which fibroblasts affect cancer susceptibility and progression via the secretion of protumoral and prometastatic cytokines.

Likewise, CAFs and ECM may affect breast tissue density, which has important clinical implication not only for cancer progression but also for mammographic screening. Indeed, in a large multiethnic study, it was shown that women of Hispanic ancestry had the highest mammographic breast density, followed by AA and EA women (84). To what extent this CAF-related phenotype is affected by differential deposition of collagen, collagen crosslinking, or regulation of interstitial pressure among the different racial groups needs to be determined using *in vivo* studies in the future.

### Cancer-Associated Adipocytes in Patients of Different Racial/Ethnic Backgrounds

The interplay between cancer cells and adipocytes has not been extensively studied. This may potentially be due to the fact that adipocytes, which represent a large portion of the healthy breast tissue, are frequently replaced by desmoplastic stroma during cancer progression. Nevertheless, cancer cells often invade the surrounding adipose tissues and such interplay may affect breast cancer outcome (85). Indeed, several studies indicate a positive correlation between cancer cell invasion into adipose breast tissue and poor patient outcome (86, 87). Recently, a microanatomical adipocyte-associated structure called crown-like structure (CLS) was described in breast TME (54). CLS are composed of macrophages surrounding dying adipocytes. A higher density of CLS was found in black compared to Caucasian and non-black Hispanic patients (54). Interestingly, CLS containing pro-inflammatory M1 macrophages are associated with worse survival in all racial groups. Thus, adipocytes may affect cancer outcome by influencing cancer behavior locally, as shown in several *in vitro* studies (88). Alternatively, adipocytes may be affecting overall inflammation at the systemic level, which is also cancer-promoting (89, 90). Since AA race is associated with higher obesity rates compared to EA (91), and obesity induces low-grade chronic inflammatory milieu, it is possible that CLSs are more frequently associated with M1 macrophages in AA than in EA patients due to obesity-induced inflammation. Indeed, obesity is not only associated with increased circulating fatty acids, but also with enrichment of chemo-attractants for immune cells into the TME (92). In particular, adipose tissue produces inflammatory cytokines such as TNF-α, interleukin (IL-6), IL-1β, and monocyte chemoattractant protein (MCP)-1. Moreover, adipocytes transdifferentiate into macrophages, which can be stimulated by fatty acids to produce inflammatory cytokines. High cytokine levels perpetuate chronic inflammation, which in turn, can promote tumor progression. Therefore, the interplay between TME and circulating cytokines may be responsible for the association of obesity with worse outcome in patients with breast cancer (93).

## Serum Cytokine Profile in Breast Cancer Patients of Different Racial/Ethnic Backgrounds

Cytokines, the signaling molecules that mediate and regulate immunity, inflammation and hematopoiesis, are the biological milieu and constitute important components of the TME associated with breast cancer (94, 95). Cytokines have been used as biomarkers for prognosis and have been associated with clinical symptoms and adverse outcomes in breast cancer (95).

Studies indicate that certain cytokine levels may be influenced by racial background of the patient. For instance, plasma levels of IL-8 and granulocyte colony-stimulating factor were elevated in AAs compared with EAs (72), and TNF-α has been reported to be higher in non-obese Mexican Americans compared with matched non-Hispanic whites (96). Moreover, it was also demonstrated that plasma levels of circulating cytokines are influenced by both age and race (97). Most studies comparing racial differences in cancer at the cytokine levels investigated only a few cytokines. The reason may be the lack of sufficient numbers of AA patients in population-based case-control studies to observe significant differences in circulating cytokines and race-specific associations between cytokines and cancer (98). Studies in various cancer types demonstrated that there are substantial racial differences in inflammation between AA and EA patients. In lung cancer for instance, certain cytokines (IL-4, IL-5, IL-8, IL-10, IFN-γ, and TNF-α) were significantly elevated among EA compared to AA patients, whereas elevated IL-1β, IL-10, and TNF-α levels were associated with lung cancer only among AA patients (98). In other studies, AA compared to EA patients appeared to have higher levels of circulating C-reactive protein [a non-specific marker of inflammation (99)], higher levels of IL-6, and reduced levels of TNF-α (100). Of note, AA and EA patients were found to have significantly different frequencies of single nucleotide polymorphisms (SNPs) in cytokine genes, which may functionally alter and explain the differences in serum cytokine concentrations (99, 100).

A recent study demonstrated that race affects inflammatory cytokine levels (IL-6 and IFN-γ) and breast cancer risk. (101). Interestingly, other studies have shown that milk from healthy black women may contain higher levels of IL-1β than from white women even when controlled for BMI (102), which strengthens the hypothesis that increased inflammation within the breast of black women compared with white women may be linked to the higher rates of early onset breast cancer in black women (103). Therefore, potential strategies to reduce racial disparities in breast cancer risk could be through interventions such as short courses of anti-inflammatory agents (102). This is further supported by preclinical results reported by Lyons et al. (104) showing that a postpartum pro-inflammatory mechanism may promote development of aggressive breast cancer. Interestingly, TAMs, one of the major contributors of pro-inflammatory cytokines, are found in higher density in breast cancer specimens from AA compared to EA patients. Among other cytokines, TAMs produce resistin, which is the main mediator of obesity associated pro-inflammatory effects in various diseases, including cancer (105). Indeed, resistin, a main inducer of IL-6, was found to be expressed at greater levels in the TME of AA than of EA patients (106), specifically in breast cancer cells. This, in turn, may promote proliferation of breast cancer cells through STAT3 activation (105).

Since cytokines operate in integrated networks, a more complete understanding will be gained with the exploration and accurate measurements of multiple cytokines simultaneously (known as cytokine patterns or signatures), using advanced proteomic technologies (107). A wide range of cytokine assays is available for accurate measurements in biological fluids, e.g., immunoassays, cytokine bioassays, multiplex bead array assays, mass spectrometry, multi-parametric flow cytometry, among others (107). However, further research using bioanalytical techniques is needed to identify patterns of cytokine expression that may serve as biomarkers in clinical research, and to determine further differences in the cytokine landscape among patients of different racial backgrounds.

## Racial/Ethnic Disparities in TME Elucidated Using High Throughput Tissue Analyses

### Disparity in TME Gene Expression Pattern

Several groups have previously compared breast cancer gene expression patterns between AA and EA patients using high throughput approaches such as RNA sequencing and found differences in signaling pathways primarily related to angiogenesis, chemotaxis and immunity (32, 55). However, the two most significantly differentially expressed genes in the breast cancer epithelia between AA and EA patients are the phosphoserine phosphatase like (PSPHL) and Beta-crystallin B2 (CRYBB2) (55).Interestingly, PSPHL and CRYBB2 are also the most differentially expressed genes in prostate cancer patients from these two ancestral backgrounds (108). In fact, the racial ancestry of 94% of breast cancer epithelia could be correctly classified based only on the expression pattern of these 2 genes. The reasons for this are not understood.

A similar prediction could be made using five genes most differentially expressed in the breast cancer stroma, PSPHL, CXCL10, CXCL11, ISG20, and GMDS. Importantly, this separation was independent of estrogen receptor expression status. Interestingly, CXCL10, CXCL11, and ISG20 are IFN-γ-regulated genes, which is consistent with the presence of interferon signature found in breast cancer from AA patients (55). There are several reasons for the presence of interferon signature in tissues from AA, including chronic inflammation and/or presence of specific mutations in immune-related genes in tumors of AA patients (109). An extensive study by the Pusztai group performed a detailed analysis of immune gene expression in a multiracial patient cohort. The authors compared expression of 14 immune metagenes (patterns of gene expression) between AA and EA tumors, and found that although the median expression of all metagenes were higher in tumors from AA, only the major histocompatibility complex-1 (MHC1) was expressed at statistically significant higher levels. After looking deeper into the differences within tumor subtypes, it became evident that ER+ but not TN breast cancers from AA had higher median expression of the MHC1 metagene. Furthermore, the tumor immune dysfunction and exclusion (TIDE) analysis, which is used to assess the function and inclusion of T cells in the TME, showed only IFN-γ to be statistically higher in AA tumors, consistent with the presence of INF-γ signature in breast TME of AA women (55). Thus, IFN-γ network appears to be a main difference in breast cancer TME between AA and EA patients.

### Disparity in Genomic Variations Affecting the TME

Racial differences in the immune TME have also been observed at a genomic level. It has been postulated and confirmed by several studies that populations with geographic ancestries that have been heavily exposed to environmental pathogens have variants in genes involved in innate immunity that protect them against infection, but negatively impact cancer incidence and progression (109). In a proof-of-principle, pilot study for example, it was shown that a Cypriot population displayed higher risk of developing cancer when there was a prior exposure to parasitic infections by *Echinococcus granulosus* (110). Such observations suggest that genomic variations may be prevalent in certain ethnic groups or patient populations of variable geographic origins, possibly as an inadvertent result of protection against local/endemic pathogens. Further evidence by Lazarus et al. (111) demonstrated that distinct SNPs patterns exist in innate immune genes in AA compared to EA patients. Likewise, Kwiatkowski et al. (111) found higher incidence of SNP variants in AA than in EA indicating that greater haplotype diversity exists within AA gene pool.

These observations collectively suggest that racial differences in transcriptomic/genomic landscape are indeed prevalent among breast cancer patients, which partially explain the intrinsic differences in the tumor microenvironment composition and disease progression.

## Can Racial/Ethnic Disparities in Breast TME Help Personalize Breast Cancer Therapy?

The rationalized targeting of the tumor microenvironment has been proposed as early as the publication of the original “Hallmarks of Cancer” by Weinberg and Hanahan (112). In this review, we add a new dimension to this premise: different racial backgrounds are associated with different tumor microenvironments, which may partly explain the disparities in disease development and progression. This premise suggests that in the era of personalized oncology and rationalized targeting of the tumor microenvironment, race should clearly be taken into account as a major determinant of TME composition. Unfortunately, successful targeting of the components of TME have proven to be challenging. For example, anti-angiogenic drug bevacizumab (humanized monoclonal anti-VEGF antibody), failed to improve overall survival in either localized or metastatic breast cancer despite promising pre-clinical results (113). The key to successful bevacizumab treatment may lie in identifying patients with the appropriate TME, which could be racially determined (114). We postulate that studying racial disparities in the context of TME may facilitate identification of novel biomarkers for tailored treatment and for development of new therapeutics that specifically target the TME in AA. An example is the targeting of TMEM function using TMEM score as a prognostic for patients who would respond. Drugs specific for inhibition of macrophages supporting the assembly and function of TMEM its associated tumor cell dissemination, such as rebastinib (115), might present an opportunity if the TMEM score is elevated in AA patients.

Although prior epidemiologic and meta-analysis studies have documented that breast cancer patients treated with neoadjuvant (NAC) vs. adjuvant (AC) chemotherapy have no difference in survival (116, 117), a recent study by Pastoriza et al. (118) which stratified patients according to race, demonstrated that black patients treated with NAC have worse distant recurrence-free survival (DRFS) compared to matched white patients. Such racial disparities may partially be due to differences in the TME between AA and EA patients, including the increased density of prometastatic TAMs and microvascular density in black compared to white patients (54, 55). Since the main cause of breast cancer morbidity is metastatic disease, in addition to shrinking tumors with cytotoxic and anti-angiogenic therapies, targeting the sites of hematogenous dissemination at TMEM doorways may modify the TME and improve overall survival (115, 119, 120). Since AA compared to EA patients have higher microvascular and macrophage density as explained above, they may also have higher density of TMEM doorways, and thus respond better to anti-angiogenic and anti-TMEM therapy. Examining racial differences in TME may identify subpopulations of patients that do not receive full clinical benefit from current standardized therapies, and can define the need for novel, alternative treatment options in such patients.

Other promising therapies targeting the TME are immunotherapies (121). Although breast cancer is generally not highly immunogenic, the response to immunotherapies may vary according to the subtype: TN breast cancer, for example, is considered as the most immunogenic subtype, whereas ER+ disease is not (122). Since AA women tend to have higher incidence of TN disease as a population, one may speculate that AA patients may benefit more from immunotherapy. It would be interesting to evaluate if there is a racial disparity in patient response to immunotherapy. TCGA RNA sequencing data show significantly greater expression of the PD-L1 gene as compared to non-TNBC (123). Further studies established a link between androgen receptor (AR) expression in breast cancer and distinct gene signatures finding that those breast cancers with a lack of AR expression and triple negative biology had shorter time to progression and decreased overall survival with significantly elevated expression for immune checkpoint inhibitors PD-1, PD-L1, and CTLA 4. AR status was found to be a prognostic marker with increased capacity for AA patients (124). These findings show promise for the potential selective use of checkpoint inhibitors in this population. The lack of AR expression in the tumor can be used as a surrogate marker for increased expression in checkpoint inhibitors as PD-L1 expression in tumors has not been shown to be a reliable biomarker in regards to durable response to therapy (125). Further studies would need to be done in order to confirm whether AR status can be used in this way and if a correlation exists between AR expression and response to anti-PD-1/PD-L1-directed treatments.

## Geographic Ancestry—The Ultimate Culprit for Disparity in TME of Breast Cancer?

**Figure 1:**
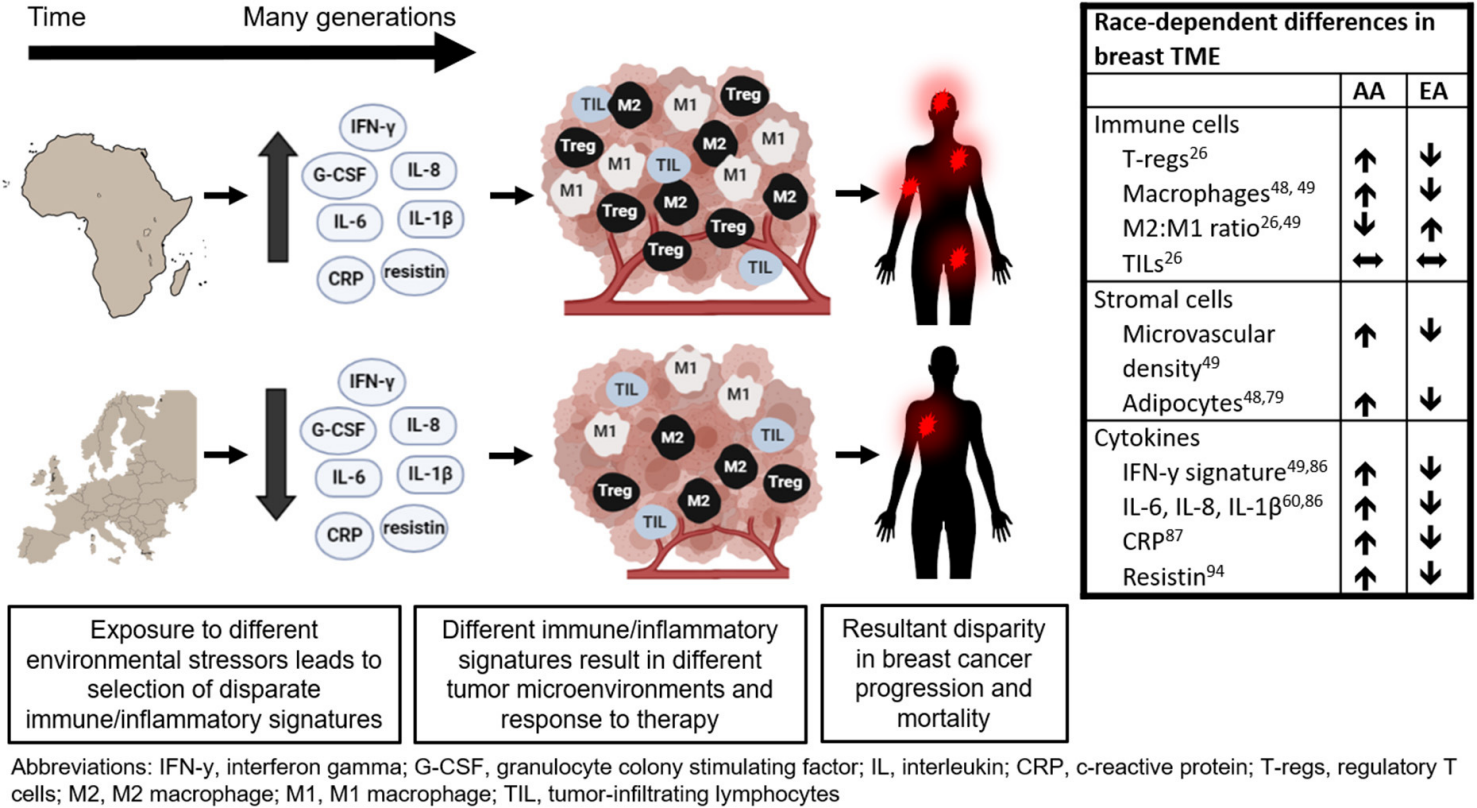
Potential link between tumor microenvironment and racial disparity in breast cancer outcome.

Disparities encountered in the TME are a part of the dynamic interplay between local and systemic factors. As discussed above, the most pronounced differences in TME are associated with inflammation and angiogenesis. African ancestry is associated with higher inflammatory gene expression and enhanced bacterial clearance likely due to pathogen-rich geographic ancestry. While aggressive immune response is beneficial in defeating pathogens prevalent in certain geographical regions, it may also be promoting pro-tumorigenic properties in the TME as an unintended consequence. These protective innate immune variants are both disproportionately distributed among racial populations and are linked with racial disparities in cancer (109). Therefore, genetic and phenotypic characteristics that developed in response to environmental stressors specific to a particular geographic ancestry region may be the underlying cause for racial disparity in TME and ultimately outcome in patients with breast cancer (Figure 1) (126).

## Conclusion

The TME is rapidly emerging as a key contributor to cancer progression, and patient outcome. The complex interplay between tumor cells and surrounding immune, vascular, and stromal components continue to be studied extensively. In this review, we highlight the racial differences in TME on cellular, molecular, and genetic levels. Furthermore, we explore systemic immune and cytokine signatures as contributors to the racial disparity in TME. The awareness of these differences and further research will lead to development of race-specific biomarkers and therapeutic targets and ultimately improved personalized cancer treatment.

## Author Contributions

GKa and MO contributed conception and design of the review. GKi, JP, GKa, and MO wrote sections of the manuscript. JS, JC, and PF provided critcial revisions to the content. All authors contributed to the article and approved the submitted version.

## Conflict of Interest

The authors declare that the research was conducted in the absence of any commercial or financial relationships that could be construed as a potential conflict of interest.
